# Early antiproteinuric effect of voclosporin in patients with LN in a real-life setting: preliminary results from the VoRLiSS (Voclosporin in Real Life Setting Study) experience

**DOI:** 10.1093/rheumatology/keaf654

**Published:** 2025-12-12

**Authors:** Luca Iaccarino, Aurora Di Gregorio, Marta Calatroni, Marco Allinovi, Alessandra Bortoluzzi, Giorgio Buscetta, Rossella De Angelis, Ginevra De Marchi, Giacomo Emmi, Pasquale Esposito, Paride Fenaroli, Cinzia Ferio, Franco Franceschini, Micaela Fredi, Francesco Gozza, Giuliana Marongiu, Giovanni Orsolini, Giulia Pazzola, Daniela Picciotto, Luca Quartuccio, Francesca Ruzzon, Domenico Santoro, Ettore Silvagni, Paola Tomietto, Filippo Vesentini, Margherita Zen, Andrea Doria, Gabriella Moroni

**Affiliations:** Rheumatology Unit, Department of Medicine-DIMED, University of Padua, Padua, Italy; Rheumatology Unit, Department of Medicine-DIMED, University of Padua, Padua, Italy; Nephrology and Dialysis Unit, IRCCS Humanitas Hospital, Milan, Italy; Department of Biomedical Sciences, Humanitas University, Milan, Italy; Nephrology, Dialysis and Transplantation Unit, Careggi University Hospital, Florence, Italy; Rheumatology Unit, Azienda Ospedaliera-Universitaria Sant’Anna di Cona, Ferrara, Italy; Department of Medical Sciences, University of Ferrara, Ferrara, Italy; Rheumatology Unit, Azienda Ospedaliera Universitaria Policlinico “Paolo Giaccone”, Palermo, Italy; Rheumatology Unit, Department of Clinical and Molecular Sciences, Polytechnic University of Marche, Ancona, Italy; Rheumatology Unit, Department of Medical Specialties, Azienda Sanitaria Universitaria Friuli Centrale, Udine, Italy; Department of Medical, Surgical and Health Sciences, University of Trieste, Trieste, Italy; Department of Internal Medicine and Medical Specialties (DIMI), University of Genoa, Genoa, Italy; Unit of Nephrology, Dialysis and Transplantation, IRCCS Ospedale Policlinico San Martino, Genoa, Italy; Nephrology and Dialysis Unit, Azienda USL-IRCCS, Reggio Emilia, Italy; Department of Clinical and Experimental Medicine, Nephrology and Dialysis, University of Messina, Messina, Italy; Rheumatology and Clinical Immunology Unit, ASST Spedali Civili and Clinical and Experimental Science Department, University of Brescia, Brescia, Italy; Rheumatology and Clinical Immunology Unit, ASST Spedali Civili and Clinical and Experimental Science Department, University of Brescia, Brescia, Italy; Rheumatology Unit, Azienda Ospedaliera Universitaria Policlinico “Paolo Giaccone”, Palermo, Italy; Nephrology, Dialysis and Transplantation Unit, Careggi University Hospital, Florence, Italy; Rheumatology Unit, AOUI Verona, Verona, Italy; Rheumatology Unit, Azienda USL-IRCCS di Reggio Emilia, Reggio Emilia, Italy; Unit of Nephrology, Dialysis and Transplantation, IRCCS Ospedale Policlinico San Martino, Genoa, Italy; Rheumatology Unit, Department of Medicine, University of Udine and Azienda Sanitaria Universitaria Friuli Centrale, Udine, Italy; Rheumatology Unit, AOUI Verona, Verona, Italy; Department of Clinical and Experimental Medicine, Nephrology and Dialysis, University of Messina, Messina, Italy; Rheumatology Unit, Azienda Ospedaliera-Universitaria Sant’Anna di Cona, Ferrara, Italy; Department of Medical Sciences, University of Ferrara, Ferrara, Italy; Internal Medicine, Clinical Immunology and Rheumatology Unit, Cattinara University Hospital, Trieste, Italy; Rheumatology Unit, Department of Medicine-DIMED, University of Padua, Padua, Italy; Rheumatology Unit, Department of Medicine-DIMED, University of Padua, Padua, Italy; Rheumatology Unit, Department of Medicine-DIMED, University of Padua, Padua, Italy; Nephrology and Dialysis Unit, IRCCS Humanitas Hospital, Milan, Italy; Department of Biomedical Sciences, Humanitas University, Milan, Italy

**Keywords:** SLE, LN, 24-h proteinuria, voclosporin, calcineurin inhibitors

## Abstract

**Objective:**

To assess efficacy of voclosporin (VCL) from a retrospective, observational, clinical-practice-based, nationwide multicentre study of patients with LN.

**Methods:**

Patients with biopsy-proven LN were enrolled from November 2023 to April 2025 from tertiary Rheumatology and Nephrology Italian Centres. Those with uncontrolled arterial hypertension and eGFR < 30 ml/min/1.73 m^2^ were excluded. Patients received oral 23.7 mg VCL BID and MMF 1 g BID. Glucocorticoid schedule followed existing recommendations for LN management. Clinical and serological data were collected at SLE diagnosis, VCL initiation and after 6, 12, 24 and 48 weeks. Complete renal response (CRR) was defined as eGFR ≥ 60 ml/min/1.73 m^2^, <20% eGFR decrease from baseline, 24-h proteinuria <0.5 g/day, no rescue therapy, prednisone ≤5 mg/day and partial renal response (PRR) as a 50% decrease of 24 h proteinuria and <20% eGFR decrease.

**Results:**

Forty-two patients from 14 centres were enrolled, 26 females (61.9%), mean age 43.1 ± 11.9 years, follow-up 6.6 ± 5.1 months. A total of 31.5% of patients achieved CRR or PRR at 6 weeks, 68.9% at 12 weeks, 83.3% at 24 weeks and 91.6% at 48 weeks of follow-up. A significant decrease in 24-h proteinuria was observed at 6 weeks (*P* = 0.006) and at all subsequent time points. A mild eGFR decrease was observed at 6 (*P* = 0.008) and 24 weeks (*P* = 0.01), but not at 48 weeks. Significant decrease was also observed in anti-dsDNA positivity at 6 (*P* = 0.0016) and 12 weeks (*P* = 0.0009), and in SLEDAI-2K after 24 (*P* = 0.008) and 48 weeks (*P* = 0.05).

**Conclusions:**

VCL may provide a valuable therapeutic option in LN management, achieving early 24 h-proteinuria response consistent with clinical trial data.

Rheumatology key messages

- LN patients treated with voclosporin displayed an early proteinuria response.
- The majority of patients achieved complete or partial renal response after 6 months of follow-up.
- Voclosporin, associated with MMF, may represent a valuable therapeutic option in LN.

## Introduction

Lupus nephritis (LN) is a severe manifestation of systemic lupus erythematosus (SLE) that can lead to end-stage kidney disease in ∼15% of cases [1–3]. Therefore, the primary goal of LN treatment is to control renal inflammation and preserve kidney function.

Voclosporin (VCL) is a novel calcineurin inhibitor (CNI) that offers several advantages over tacrolimus and ciclosporin. VCL has a stable pharmacokinetic profile, eliminating the need for intensive drug monitoring, and a lower impact on lipid and glucose metabolism. Moreover, VCL does not affect plasma concentrations of mycophenolic acid in patients receiving mycophenolate mofetil (MMF).

In Phase 2 (AURA-LV) and Phase 3 (AURORA) randomized controlled trials [4, 5], VCL in combination with MMF and very low-dose corticosteroids achieved higher renal response rates at 24 and 52 weeks compared with MMF and corticosteroids alone. Based on these findings, VCL was approved in January 2021 by the US FDA, and in September 2022 by the EMA for LN treatment, and included in the 2023 update of EULAR recommendations for SLE [6].

The AURORA 2 extension study [7] confirmed long-term safety and efficacy up to 3 years, showing no new safety signals or increased risk of infections, malignancies, hypertension or renal deterioration. However, its efficacy and safety still need to be confirmed in real-world clinical settings. This study aims to evaluate VCL efficacy and safety in a nationwide, multicentre cohort of LN patients.

## Methods

VoRLiSS is an observational multicentre study, involving prospectively followed-up cohorts in tertiary Italian centres. Participating physicians included rheumatologists, immunologists and nephrologists.

We included patients with SLE classified by ACR 1982, SLICC or EULAR/ACR criteria, with active, biopsy-proven LN. Exclusion criteria were uncontrolled arterial hypertension and eGFR <30 ml/min/1.73 m^2^.

Patients received VCL 23.7 mg BID (three 7.9 mg softgel capsules twice daily) plus MMF 1 g BID, per clinical practice.

Glucocorticoid schedule (pulses, oral administration and tapering) followed routine practice based on 2019 EULAR/ERA-EDTA and/or 2024 KDIGO guidelines [8, 9]. Data were entered into an anonymized *Ad hoc* database.

The following clinical and laboratory variables were collected at SLE diagnosis, at VCL initiation and at 6, 12, 24 and 48 weeks:

SLEDAI-2K, CLASI (activity/damage for skin), DAS28-CRP (for joint), SLE-DAS, PGA (0–3), anti-double-stranded (ds)DNA antibodies (IFI, Farr and/or ELISA), C3/C4, complete blood cell count, 24-h proteinuria, serum creatinine, eGFR (measured with CKD EPI equation), urine analysis, protein electrophoresis, total cholesterol, triglycerides, fasting glucose, venous pH, blood pressure, RAS inhibitors, prednisone dose and concomitant immunosuppressants.

Data consistency was checked centrally, and centres corrected missing or inconsistent information. Outcomes were complete renal response (CRR), defined as (1) 24 h proteinuria <0.5 g/day; (2) eGFR ≥ 60 ml/min/1.73 m^2^, and no eGFR decrease of >20% from baseline; (3) no rescue therapies for LN; (4) daily prednisone ≤5 mg, and partial renal response (PRR), defined as (1) 50% decrease of baseline 24 h proteinuria or proteinuria <3.5 g/day in nephrotic patients; (2) no eGFR decrease of >20% from baseline.

Extra-renal parameters evaluated included SLEDAI-2K, SLE-DAS, CLASI and DAS28-CRP.

Safety assessment included all adverse events (AEs), severe and non-severe, at each visit. Discontinuation of VCL with reasons was also documented.

### Statistical analysis

Data are expressed as mean ± standard deviation (S.D.) due to their parametric distribution. Therefore, continuous data were compared with repeated measures ANOVA with Bonferroni’s *post hoc* test. SPSS software (version 28.0, Chicago, IL, USA) was used for statistics, setting statistical significance at *P* < 0.05.

Associations between potential predictors and treatment response at 12 weeks, considered as early response, were assessed using the *χ*^2^ test or Fisher’s exact test, as appropriate, for categorical variables, and the *t*-test for continuous variables with parametric distribution. A *P*-value <0.05 was considered statistically significant.

### Ethics

The study was submitted to the University of Padua Ethics Committee (3806/AO/16) and was carried out in accordance with the Declaration of Helsinki. Written informed consent was obtained from all patients.

## Results

### Demographic and clinical features

Forty-two patients from 14 referral centres (nine rheumatology and five Nephrology units) were enrolled, 26 females (61.9%), mean age 43.1 ± 11.9 years at VCL initiation. The mean age at diagnosis of LN was 30 ± 12.8 years, and 90.2% of patients were Caucasians. Lag time between diagnosis of SLE and VCL initiation was 119.45 ± 111.84 months, and between last kidney biopsy and the start of VCL was 27.2 ± 33.6 months. At VCL initiation, the mean eGFR was 96.5 ± 25.3 ml/min (eGFR < 90 ml/min in 30.9% of patients), 24-h proteinuria was 2.54 ± 1.59 g (nephrotic syndrome in 35.7% of patients) and seven patients (16.7%) had arterial hypertension, defined as ≥140/90 mmHg. C3 and C4 serum levels were low in 26.2% and 28.5% of patients, respectively; anti-dsDNA antibodies were positive in 64.3% of patients, and the mean SLEDAI-2K was 14.95 ± 5.91. The mean follow-up after VCL initiation was 6.6 ± 5.03 months, with 24 patients achieving at least 24 weeks of follow-up. Demographic, clinical and serological characteristics at SLE diagnosis and at VCL initiation (baseline) are reported in Table 1.

**Table 1:** Characteristics at SLE diagnosis and voclosporin initiation of 42 patients with LN.

Data from SLE diagnosis to voclosporin initiation	
Demographic characteristics	
Females, *n* (%)	26 (61.9)
Age at diagnosis, years, mean ± S.D.	35 ± 14.7
Caucasian, *n* (%)	37 (90.2)
Asian, *n* (%)	3 (7.1)
African, *n* (%)	2 (4.7)
Clinical manifestations	
Musculoskeletal, *n* (%)	34 (80.9)
Renal, *n* (%)	33 (78.6)
Number of previous LN flares, mean ± S.D.	2.0 ± 0.7
Cutaneous, *n* (%)	20 (47.6)
Haematological, *n* (%)	19 (45.2)
Constitutional, *n* (%)	18 (42.9)
Serositis, *n* (%)	12 (28.6)
Serology	
ANA, *n* (%)	42 (100)
Anti-dsDNA, *n* (%)	32 (76.2)
Anti-Sm, *n* (%)	14 (33.3)
Anti-U1RNP, *n* (%)	12 (28.6)
APLs, *n* (%)	11 (26.2)
Treatments	
HCQ, *n* (%)	37 (88.1)
MMF, *n* (%)	37 (88.1)
Belimumab, *n* (%)	19 (45.2)
CYC, *n* (%)	12 (28.6)
AZA, *n* (%)	11 (26.2)
Rituximab, *n* (%)	13 (30.9)
Tacrolimus, *n* (%)	10 (23.8)
Ciclosporin A, *n* (%)	8 (19.1)
MTX, *n* (%)	7 (16.7)
Anifrolumab, *n* (%)	1 (2.4)

Data at voclosporin initiation	
Demographic characteristics	
Age, years, mean ± S.D.	45.1 ± 12.1
Disease duration, years, mean ± S.D.	10.2 ± 9.2
Months between renal biopsy and voclosporin, mean ± S.D.	27.2 ± 33.6
New onset LN, *n* (%)	9 (21.4)
Follow-up, months, mean ± S.D.	6.6 ± 5.1
Renal manifestations	
Kidney biopsy data available, *n* (%)	39 (92.8)
Class, *n* (%)	
II	3 (7.1)
III	9 (21.4)
III/V	4 (9.5)
IV	7 (16.7)
IV/V	8 (19.1)
V	8 (19.1)
Asymptomatic proteinuria, *n* (%)^a^	24 (57.1)
Asymptomatic proteinuria and haematuria, *n* (%)	5 (11.9)
Nephrotic syndrome, *n* (%)^b^	15 (35.7)
Nephritic syndrome, *n* (%)^c^	3 (7.1)
Arterial hypertension, *n* (%)	7 (16.7)
Baseline extra-renal manifestations	
Musculoskeletal, *n* (%)	11 (26.1)
Cutaneous, *n* (%)	7 (16.7)
Serositis, *n* (%)	2 (4.8)
Haematological, *n* (%)	13 (30.9)
Antiphospholipid syndrome, *n* (%)	3 (7.1)
Serology at baseline	
Anti-dsDNA, *n* (%)	27 (64.3)
Anti-Sm, *n* (%)	8 (19.1)
Anti-U1RNP, *n* (%)	8 (19.1)
Anti-SSA, *n* (%)	6 (14.3)
Anti-P ribosomal, *n* (%)	2 (4.8)
Anti-C1q, *n* (%)	1 (2.4)
Concomitant medications	
Prednisone, *n* (%)	42 (100)
MMF, *n* (%)	42 (100)
HCQ, *n* (%)	29 (69.4)
Angiotensin converting enzyme inhibitors, *n* (%)	29 (69.4)
Angiotensin receptor blockers, *n* (%)	8 (19.1)
Belimumab, *n* (%)	7 (16.7)

Arterial hypertension: blood pressure ≥140/90 mmHg.

a Asymptomatic proteinuria: proteinuria between 0.5 and 3.5 g/day.

b Nephrotic syndrome: 24 h proteinuria >3.5 g/day and serum albumin <3.0 g/dl.

c Nephritic syndrome: acute deterioration of kidney function, severe haematuria and arterial hypertension.

### Efficacy of VCL

Variation of different clinical, biochemical, serological and urinary variables during the follow-up after the start of VCL therapy is reported in detail in Table 2. Considering the main outcomes, 31.5% of 38 patients achieved CRR or PRR after 6 weeks, 68.9% of 29 patients at 12 weeks, 83.3% of 24 patients at 24 weeks and 91.6% of 12 patients at 48 weeks of follow-up. Overall, 31 out of 38 (81.6%) patients achieved a renal response, either PRR or CRR, at the last available follow-up. Factors associated with overall renal response are reported in Table 3. Notably, no difference in overall response was observed when patients were stratified according to the timing of VOC initiation with respect to renal biopsy (within or after 12 months, 13/16 vs 18/22, *P* = 1.0; within or after 24 months, 20/26 vs 11/12, *P* = 0.27); similar findings were obtained when patients were stratified according to the previous use of other CNIs (overall renal response in CNI-exposed patients: 14/16 vs 17/22 in non-exposed patients, *P* = 0.67). No correlation between the number of previous treatments for LN and overall response was observed (Spearman’s rho coefficient = 0.111, *P* = 0.506).

**Table 2:** Complete and partial renal response and variation of clinical, biochemical, serological and urinary variables at different timepoints.

	T0	T1 (6 weeks)	*P* ^*^	T2 (12 weeks)	*P* ^*^	T3 (24 weeks)	*P* ^*^	T4 (48 weeks)	*P* ^*^
Number of patients	42	38		29		24		12	
Complete renal response	0	7 (18.4)	–	11 (37.9)	–	11 (45.8)	–	5 (41.6)	**–**
Partial renal response	0	5 (13.1)	**–**	9 (31.03)	–	9 (37.5)	–	6 (50)	–
SLEDAI-2K (mean ± S.D.)	9.6 ± 5.3	7.7 ± 4.6	0.21	7.0 ± 4.4	0.19	6.4 ± 5.7	**0.008**	6.4 ± 5.7	**0.05**
SLE-DAS (mean ± S.D.)	14.9 ± 5.9	12.2 ± 4.8	0.084	8.9 ± 5.2	**0.0007**	9.9 ± 8.3	**0.019**	7.8 ± 4.2	**<0.0001**
PGA (mean ± S.D.)	1.29 ± 0.91	1.19 ± 0.79	0.90	0.98 ± 0.68	0.50	0.72 ± 0.57	**0.002**	0.72 ± 0.56	**0.016**
Anti-dsDNA positive, *n* (%)	27 (64.3)	11 (28.9)	**0.0016**	7 (24.1)	**0.0009**	10 (41.6)	0.053	5 (41.6)	0.16
C3 mg/dl (mean ± S.D.)	0.93 ± 0.28	1.06 ± 0.29	**0.01**	1.04 ± 0.28	0.164	0.97 ± 0.24	0.58	0.98 ± 0.24	0.55
C4 mg/dl (mean ± S.D.)	0.33 ± 0.15	0.23 ± 0.13	0.29	0.23 ± 0.14	0.421	0.2 ± 0.07	0.24	0.19 ± 0.08	0.35
Creatinine mg/dl (mean ± S.D.)	0.87 ± 0.34	0.98 ± 0.44	**0.003**	0.9 ± 0.48	0.058	0.9 ± 0.36	**0.004**	0.9 ± 0.1	0.06
eGFR, ml/min/1.73 m^2^ (mean ± SD)	96.5 ± 25.3	85.5 ± 27.5	**0.008**	87.9 ± 30.5	0.338	89.3 ± 21.8	**0.01**	87.9 ± 17.6	0.064
24-h proteinuria, g (mean ± S.D.)	2.54 ± 1.59	1.6 ± 1.4	**0.006**	1.02 ± 0.88	**<0.001**	1.02 ± 0.9	**<0.001**	0.8 ± 0.65	**<0.001**
Active urine sediment, *n* (%)	20 (47.6)	13 (30.9)	0.224	12 (28.5)	0.603	8 (19.4)	0.210	2 (4.76)	0.054
SBP, mmHg (mean ± S.D.)	123.9 ± 12.02	120.9 ± 11.4	0.258	131.7 ± 17.4	**0.042**	126.4 ± 13.01	0.438	128.2 ± 11.65	0.277
DBP, mmHg (mean **±** S.D.)	77.97 ± 9.2	80.26 ± 7.03	0.05	82.64 ± 12.76	0.409	80.09 ± 5.45	0.241	83.9 ± 5.56	0.394
PDN daily dosage, mg (mean ± S.D.)	11.5 ± 13.7	8.5 ± 8.16	0.119	5.6 ± 4.6	0.058	5.6 ± 4.6	0.589	5.8 ± 6.5	0.296
PDN ≤5 mg/day, *n* (%)	18 (42.8)	22 (57.8)	–	18 (62.1)	–	15 (62.5)	–	10 (83.3)	–
PDN discontinued	4 (9.5)	4 (10.5)	–	4 (13.7)	–	4 (16.6)	–	1 (8.3)	–
DAS28-CRP (mean ± S.D.)	2.65 ± 1.43	2.62 ± 1.27	–	NA	–	NA	–	2.28 ± 0.72	–
CLASI activity (mean ± S.D.)	1.86 ± 5.3	1 ± 2.5	–	0.41 ± 1.16	–	0.25 ± 0.86	–	0.37 ± 0.74	–

CLASI, Cutaneous Lupus Erythematosus Disease Area and Severity Index; DAS28 CRP, Disease Activity Score 28; DBP, diastolic blood pressure; NA, not available; PDN, prednisone; PGA, Physician Global Assessment (0–3 scale); SLEDAI-2K, Systemic Lupus Erythematosus Disease Activity Index 2000; SLE-DAS, systemic lupus erythematosus disease activity state; SBP, systolic blood pressure.

* *P* refers to the comparison between T0 and T1 (38 patients), T0 and T2 (29 patients), T0 and T3 (24 patients), T0 and T4 (12 patients), *P* values <0.05 are reported in bold. DAS28-CRP was available in four patients at T1 and three patients at T4; CLASI was evaluated in two patients at T0, T1, T3 and T4 (*P*-value not calculated for low number of cases).

**Table 3:** Characteristics of patients achieving or not complete or partial renal response at last available observation.

Demographic and clinical characteristics	Responders (31 pts)	Non-responders (7 pts)	*P*-value
Females, *n* (%)	17 (54.8)	6 (85.7)	0.209
Age at diagnosis, years, mean ± S.D.	35.89 ± 14.7	29.37 ± 15.43	0.3
Age, years, mean ± S.D.	46.43 ± 12.37	41.87 ± 10.87	0.37
Disease duration, years, mean ± S.D.	10.54 ± 9.49	12.5 ± 11.1	0.635
Ethnicity, Caucasian, *n* (%)	27 (87.1)	6 (85.7)	1
New onset LN, *n* (%)	6 (25)	2 (50)	0.55
Number of previous renal flares, mean ± S.D.	1.83 ± 1.85	1.5 ± 1.73	0.74
Anti-dsDNA positive, *n* (%)	21 (67.7)	4 (57.1)	0.672
C3 serum levels, mg/l	101	88.7	0.445
C4 serum levels, mg/l	28.39	17.43	0.374
SLEDAI-2K, mean ± S.D.	8.39 ± 4.88	11.14 ± 5.75	0.199
SLE-DAS, mean ± S.D.	14.53 ± 6.94	13.13 ± 4.57	0.646
SLICC damage index, mean ± S.D.	1.33 ± 1.15	1.66 ± 2.52	0.645
PDN daily dose, mg	7.87 ± 7.48	17.89 ± 19.78	0.232
RAAS users, *n* (%)	31 (100)	7 (100)	–
Renal manifestations			
Asymptomatic proteinuria, *n* (%)^a^	18 (58.06)	5 (71.4)	0.681
Nephrotic syndrome, *n* (%)^b^	14 (45.2)	1 (14.3)	0.209
Nephritic syndrome, *n* (%)^c^	86 (83.9)	3 (42.9)	0.146
CKD, *n* (%)	0 (100)	0 (100)	–
Serum creatinine, mg/dl, mean ± S.D.	0.9 ± 0.35	0.9 ± 0.39	0.98
eGFR, ml/min/1.73 m^2^, mean ± S.D.	93.62 ± 22.9	94.83 ± 33.62	0.913
24 h proteinuria, g, mean ± S.D.	2.45 ± 1.47	1.94 ± 1.08	0.424
Hypertension, *n* (%)	14 (45.2)	3 (42.9)	1
Proliferative LN at biopsy, *n* (%)	21 (72.4)	5 (71.4)	1
Membranous LN at biopsy, *n* (%)	16 (55.2)	3 (42.9)	0.684
Treatments prior to VCL initiation (ever)			
HCQ, *n* (%)	27 (87.1)	7 (100)	1
MMF, *n* (%)	27 (87.1)	7 (100)	1
Belimumab, *n* (%)	14 (45.2)	3 (42.9)	1
CYC, *n* (%)	10 (32.3)	1 (14.3)	0.648
AZA, *n* (%)	8 (25.8)	2 (28.6)	1
Rituximab, *n* (%)	10 (29)	2 (28.6)	0.650
Tacrolimus, *n* (%)	11 (35.5)	2 (28.6)	1
Ciclosporin A, *n* (%)	8 (25.8)	0	0.307

a Asymptomatic proteinuria: proteinuria between 0.5 and 3.5 g/day.

b Nephrotic syndrome: 24 h proteinuria >3.5 g/day and serum albumin <3.0 g/dl.

c Nephritic syndrome: acute deterioration of kidney function, severe haematuria and arterial hypertension.

A significant decrease in 24-h proteinuria was observed as early as 6 weeks after VCL initiation (*P* = 0.006), and during all the subsequent timepoints. Significant decrease was also observed for anti-dsDNA positivity at 6 weeks (*P* = 0.0016) and 12 weeks (*P* = 0.0009) of follow-up; C3 levels increased at 6 weeks (*P* = 0.01). Both SLEDAI-2K and PGA significantly decreased after 24 (*P* = 0.008 and *P* = 0.002, respectively) and 48 weeks (*P* = 0.05 and *P* = 0.016, respectively), and a significant improvement was observed in SLE-DAS at 12 (*P* = 0.0007), 24 (*P* = 0.019), and 48 weeks of follow-up (*P* < 0.0001). No significant changes were observed in C4, active urine sediment, DAS28 CRP and CLASI.

Prednisone daily dose decreased from 11.5 ± 13.7 mg/day at baseline to 5.6 ± 4.6 mg at 12 weeks and 5.8 ± 6.5 mg at 48 weeks, with 22/29 (75.8%) patients achieving prednisone dose ≤5 mg/day at 12 weeks, 19/24 (79.1%) at 24 weeks and 11/12 (91.6%) at 48 weeks.

We also explored potential predictors of CRR at 12 weeks, demonstrating that higher proteinuria increased the risk of non-response, while other baseline factors—including initial prednisone dose, LN class or prior therapeutic failures (including biologics)—had no significant effect (Supplementary Table S1).

### Safety of VCL

A decrease of eGFR was observed at 6 (*P* = 0.008) and 24 weeks (*P* = 0.01); however, the decrease was mild, <20% from baseline, and was not present at 12 weeks. At 48 weeks, eGFR mean values were stable compared with previous timepoints, although the progressively smaller sample size may have reduced the statistical power to detect a significant difference. Systolic blood pressure values significantly increased at 12 weeks (*P* = 0.042), but then normalized. No patients had eGFR <30 ml/min/1.73 m^2^ at baseline; five patients had an eGFR between 30 and 60 ml/min at VLC initiation: in four of them, eGFR remained stable throughout follow-up, while the remaining patient showed an improvement from 34 to 59 ml/min. In three patients with baseline eGFR values around 60 ml/min, a progressive increase of >10 ml/min was observed. In two patients, VLC dosage was reduced at 6 weeks due to a significant decline in eGFR, and in one patient VCL was stopped due to eGFR decrease from 114 at baseline to 55 ml/min at 6 weeks. During follow-up, five patients reduced the MMF dose due to non-severe gastrointestinal side effects (three patients at 6 weeks and two at 24 weeks; mean MMF dose at 6 weeks: 1581 ± 485 mg; at 24 weeks: 1522 ± 607 mg). Among these patients, two were able to return to the full MMF dose during subsequent follow-up (mean MMF dose at 48 weeks: 1625 ± 570 mg).

Interestingly, no extra-renal flares were observed. One patient experienced an increase in proteinuria at 6 months, which was managed by increasing the prednisone dose (from 5 to 25 mg/day), without any changes in immunosuppressive therapy.

On the other hand, no major safety concerns, particularly no deaths and no life-threatening or requiring hospitalization adverse events occurred during follow-up. In particular, during VOC exposure, no severe infections (bacterial, viral or fungal) occurred, and no cases of herpes zoster virus infection were observed. Furthermore, no significant differences were found regarding the metabolic profile of patients (total cholesterol and triglycerides, fasting glucose, venous pH).

## Discussion

This multicentre, nationwide study provides new evidence on the efficacy of VCL in LN [4, 5, 7, 10]. When added to standard therapy with MMF and corticosteroids, VCL induced a rapid reduction in 24-h proteinuria, evident by week 6, with CRR or PRR achieved in ∼70% of patients at week 12 and >80% and 90% at weeks 24 and 48, respectively. These findings support its role in early and sustained disease control in real-life practice. However, the results are preliminary and based on a small, mainly Caucasian cohort with a relatively low prevalence of nephrotic syndrome (35.7%).

To date, real-world data on VCL are limited to case reports [11, 12]; this is, to our knowledge, the first observational study assessing its efficacy and safety. Real-world studies better reflect the complexity of clinical practice, thus our data complement trial-based evidence, broadening the generalizability of VCL efficacy.

One of the key findings of the present study is the rapid and significant decrease in 24-h proteinuria observed as early as 6 weeks after initiating VCL therapy. The early proteinuria decline aligns with VCL’s pharmacodynamic profile, driven by calcineurin inhibition and podocyte stabilization, and is consistent with, yet earlier than, reductions reported in the AURA-LV and AURORA trials, where changes were evident from week 24 onward. These results are in line with the AURA-LV and AURORA trials [4, 5, 7], but show an even earlier reduction in proteinuria, already evident at week 6 compared with week 24 in the trials.

Notably, as reported in Table 1, only 21.4% of patients initiated VCL immediately after renal biopsy (new-onset LN); therefore, the majority of patients received the drug later, implying that they had failed previous treatments and still had active nephritis. However, our data suggest that prior therapeutic failures, including conventional and biologic agents, did not reduce the likelihood of an early response to VCL, indicating its potential efficacy in refractory disease. Conversely, as expected, higher baseline proteinuria delayed early response, but not overall likelihood of response.

Our results also revealed a significant decrease in anti-dsDNA antibody positivity at early timepoints, which may reflect an immunomodulatory effect of VCL combined with MMF on systemic disease activity. This is further supported by a reduction in the SLEDAI-2K score at 24 and 48 weeks, although no significant changes were observed in complement levels (particularly C4) or extra-renal organ-specific activity measures such as CLASI and DAS28 CRP. These findings might suggest that the impact of VCL may be more pronounced at the level of renal disease activity rather than other organ involvement. Unlike other drugs that target B lymphocytes (i.e. belimumab, rituximab) [13–16], VCL mechanism of action targets T cell-mediated inflammation. Nonetheless, it should be emphasized that the evaluation of the efficacy of VCL in extra-renal manifestations was not the principal aim of this study, and the number of patients exhibiting cutaneous and joint involvement was small, thereby limiting the strength of any conclusions that may be drawn in this regard.

From a safety perspective, no major concerns were reported. VCL was discontinued in one patient due to a significant eGFR reduction, while a mild, transient decrease (<20% from baseline) was observed in some patients after a few weeks. This reversible decline is likely attributable to the known vasoconstrictive effect of CNIs, consistent with previously published safety data on VCL [7, 17, 18]. On the other hand, data from this study reinforce the need for regular monitoring of renal function in patients treated with VCL, similarly to other CNIs. Longer follow-up and, when feasible, per-protocol repeated biopsies are needed to fully assess the renal and metabolic safety of VCL, especially in high-risk populations.

This study has strengths and limitations. Strengths include the multicentre design and the standardized data collection, and the standardized clinometric measures for the evaluation of disease activity. The principal limitation of this study lies in the small number of patients with 48 weeks of follow-up, which precludes definitive conclusions. Other limitations include the retrospective nature of the analysis which limits causal inference, the short duration of the follow-up, the absence of a control group, and the fact that more than half of the patients presented with asymptomatic proteinuria at baseline, resulting in a low prevalence of nephrotic syndrome, which might prevent definitive conclusions regarding treatment efficacy.

## Conclusions

In conclusion, our real-life, multicentre experience preliminarily confirms the early efficacy of VCL in reducing proteinuria in patients with LN, highlighting that previous therapeutic failures do not limit the likelihood of response to VCL, whereas higher baseline proteinuria primarily affects the speed of response. Therefore, VCL represents a valuable therapeutic option for managing active LN, particularly in patients for whom early proteinuria control is a priority. From a safety point of view, regular monitoring of eGFR is recommended, especially in patients with longer disease course.

## Supplementary Material

keaf654_Supplementary_Data

## Data Availability

There are no additional unpublished data from this study to share.
