# Supplementary material for: Early antiproteinuric effect of voclosporin in patients with LN in a real-life setting: preliminary results from the VoRLiSS (Voclosporin in Real Life Setting Study) experience
Source: Rheumatology (Oxford). 2025 Dec 12;65(1):keaf654. doi: 10.1093/rheumatology/keaf654 (PMC12784457; doi:10.1093/rheumatology/keaf654)
Supplement: keaf654_Supplementary_Data [file keaf654_supplementary_data.docx]

**Supplementary Table S1. Characteristics of patients achieving or not complete renal response within 12 weeks after voclosporin initiation.**

| Demographic and clinical characteristics | Early responders  (11 pts) | Non-early responders  (18 pts) | P value |
| --- | --- | --- | --- |
| Females, n (%) | 4 (36.4) | 14 (77.7) | 0.05 |
| Age at diagnosis, years, mean±SD | 32.0±13.4 | 33.3±13.6 | 0.802 |
| Age, years, mean±SD | 44.5±11.8 | 43.7±11.7 | 0.851 |
| Disease duration, years, mean±SD | 12.5±8.4 | 10.4±10.6 | 0.573 |
| Ethnicity, Caucasian, n (%) | 9 (81.8) | 15 (83.3) | 0.917 |
| New onset LN, n (%) | 3 (27.3) | 9 (50) | 0.59 |
| Number of previous renal flares, mean±SD | 2.5±2.3 | 1.3±1.5 | 0.154 |
| Anti-dsDNA positive, n (%) | 9 (81.8) | 11 (61.1) | 0.412 |
| C3 serum levels, mg/L | 0.89±0.38 | 0.86±0.34 | 0.86 |
| C4 serum levels, mg/L | 0.19±0.10 | 0.33±0.41 | 0.302 |
| SLEDAI-2K, mean±SD | 8.4±5.3 | 9.8±5.8 | 0.514 |
| SLE-DAS, mean±SD | 12.6±9.9 | 15.8±5.8 | 0.355 |
| SLICC damage index, mean±SD | 1.1±0.4 | 1.6±2.1 | 0.544 |
| PDN daily dose, mg | 5.64 7.1 | 10.79 12.45 | 0.222 |
| RAAS users, n (%) | 9 (81.8) | 15 (83.3) | 0.917 |
| Renal manifestations | |  |  |
| Asymptomatic proteinuria, n (%) * | 8 (72.7) | 9 (50) | 0.273 |
| Nephrotic syndrome, n (%)** | 1 (9) | 7 (38.8) | 0.110 |
| Nephritic syndrome, n (%)*** | 1 (9) | 2 (11.1) | 1.000 |
| CKD, n (%) | 2 (18.1) | 1 (5.5) | 0.539 |
| Serum creatinine, mg/dL, mean±SD | 0.92±0.27 | 0.86±0.38 | 0.646 |
| eGFR, ml/min/1,73m^2^, mean±SD | 95.7±22.2 | 96.5±25.5 | 0.930 |
| 24h proteinuria, gr, mean±SD | 1.29±0.89 | 2.87±1.4 | **0.005** |
| Hypertension, n (%) | 7 (63.6) | 7 (38.8) | 0.264 |
| Proliferative LN at biopsy, n (%) | 7 (63.6) | 14 (77.7) | 0.381 |
| Membranous LN at biopsy, n (%) | 9 (81.8) | 7 (38.8) | 0.061 |
| Treatments prior to VCL initiation (ever) |  |  |  |
| Hydroxychloroquine, n (%) | 9 (81.8) | 16 (88.8) | 0.622 |
| Mycophenolate Mofetil, n (%) | 9 (81.8) | 16 (88.8) | 0.622 |
| Belimumab, n (%) | 3 (27.3) | 11 (61.1) | 0.128 |
| Cyclophosphamide, n (%) | 5 (45.5) | 4 (22.2) | 0.237 |
| Azathioprine, n (%) | 3 (27.3) | 4 (22.2) | 0.758 |
| Rituximab, n (%) | 6 (54.6) | 1 (5.5) | 0.202 |
| Tacrolimus, n (%) | 4 (36.4) | 5 (27.7) | 0.694 |
| Cyclosporine A, n (%) | 4 (36.4) | 4 (22.2) | 0.433 |

SD: standard deviation; LN: lupus nephritis; *asymptomatic proteinuria: proteinuria between 0.5 to 3.5g/day; **nephrotic syndrome: 24h proteinuria >3.5 g/day and serum albumin <3.0g/dl; ***nephritic syndrome: acute deterioration of kidney function, severe hematuria, and arterial hypertension.
